# Supplementary material for: Bioinformatics and Genomic Analyses of the Suitability of Eight Riboswitches for Antibacterial Drug Targets
Source: Antibiotics (Basel). 2022 Aug 31;11(9):1177. doi: 10.3390/antibiotics11091177 (PMC9495176; doi:10.3390/antibiotics11091177)
Supplement: Supplementary file 1 [file antibiotics-11-01177-s001.zip › antibiotics-1854209-supplementary.pdf]

Supplementary section

Bioinformatics and Genomic Analyses of the Suitability of Eight Riboswitches for Antibacterial Drug Targets

Nikolet Pavlova and Robert Penchovsky *

Department of Genetics, Faculty of Biology, Sofia University "St. Kliment Ohridski", 8 Dragan Tzankov Blvd., 1164 Sofia, Bulgaria

* Correspondence: robert.penchovsky@hotmail.com

It is necessary to assemble a collection of nucleotide sequences to perform the precise analysis of each riboswitch in individual bacteria of a single riboswitch class. They are available in several databases, such as Rfam and NCBI. The collected data is arranged in special Fasta files, and they can be subjected to subsequent bioinformatics analyses like multiple alignments and blast analysis in this format.

Table S1. PreQ1 riboswitches in different bacteria. The table contains the sequences of PreQ1 riboswitch in 6 pathogenic bacteria.

Sequence ID	Sequence Length	Sequence Description	Nucleotide Sequence
AE017334	45	PreQ1 riboswitch (*Bacillus anthracis*)	AATAACGTGGTTCGAAACCATCCCACGTAAAAAAACTAAGGAGAT
CP001056	46	PreQ1 riboswitch (*Clostridium botulinum*)	AATTGCCTAGTTTTTTATAGAGGGATGGTCTTCGAACCTCTCCCAC
AM180355	44	PreQ1 riboswitch (*Clostridium difficile*)	TCTGTACTTGTTCGAGAACTCCAAGTATAAAAAACTAAGGAATT
AE016830	43	PreQ1 riboswitch (*Enterococcus faecalis*)	CTCGGACTGGTTCGGAAACTTCCCAGAATAAAAAACTAAGTATCT
CP003583	28	PreQ1 riboswitch (*Enterococcus faecium*)	ACTTCCCAGAATAAAAAACTAAGTATCT
AE004969	45	PreQ1 riboswitch (Neisseria_gonorrhoeae)	CGCCCCGTGGTTCGAAAACCTCCCACACTAAAAAACTAAGGAAAC

The Multiple alignments provide information about the regions of the sequences that are similar and highlight the differences. By comparing the sequences of the PreQ 1 riboswitch in individual bacteria, we can find identical regions and thus select suitable parts for either a narrow-spectrum preparation or a broad-spectrum one that affects more than 1 type of bacteria. It is possible to be done by the programs Clustal X and MEGA.

After selecting the specific region, which can be targeted by designed antisense oligonucleotide or other compounds, it is necessary to do a BLAST analysis. BLAST is a Basic Local Alignment Searching Tool. That analysis could test if the selected region is found in the Human genome or the bacteria from the human gut flora. Suppose the selected part of the sequence is found in humans or bacteria with a necessary fore for gastrointestinal health. In that case, the process again starts from the multiple alignments, intending to find another target in the pathogenic bacterial riboswitch sequence.

Using the data obtained after the series of analyses, we select regions of a bacterial PreQ 1 riboswitch in one or more bacteria that is/are not found in humans and are not present in human-beneficial bacteria. This means that we will ensure the possibility of their targeting without harm to the human body.

Figure S1. Visualization of the Multiple alignments with ESPript 3.0. ESPript 3.0. (*Easy Sequencing in PostScript'*) is a program which renders sequence similarities and secondary structure information from aligned sequences for analysis and publication purposes. (https://espript.ibcp.fr/ESPript/ESPript/)

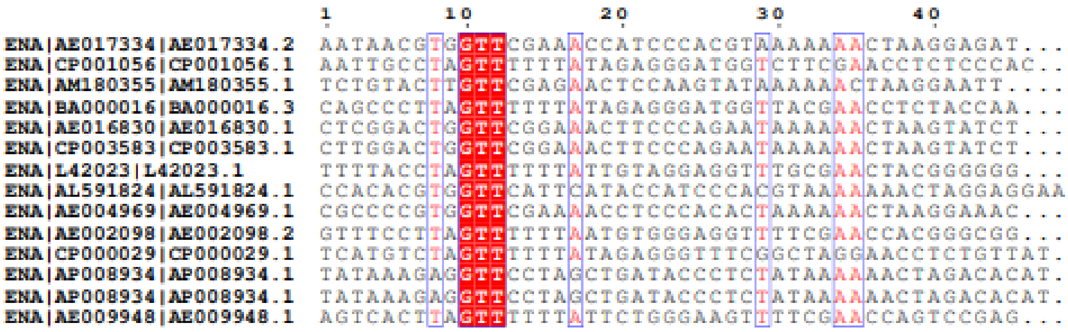

The choice of a motif and the precise work afterward on the design of the antisense oligonucleotides with which to treat it are not the subject of this article.

Figure S2. BLASTN analyses for the presence of PreQ 1 riboswitch in the Human genome. No significant similarity is found between the human genome and the *Bacillus anthracis*'s PreQ1 riboswitch sequence.

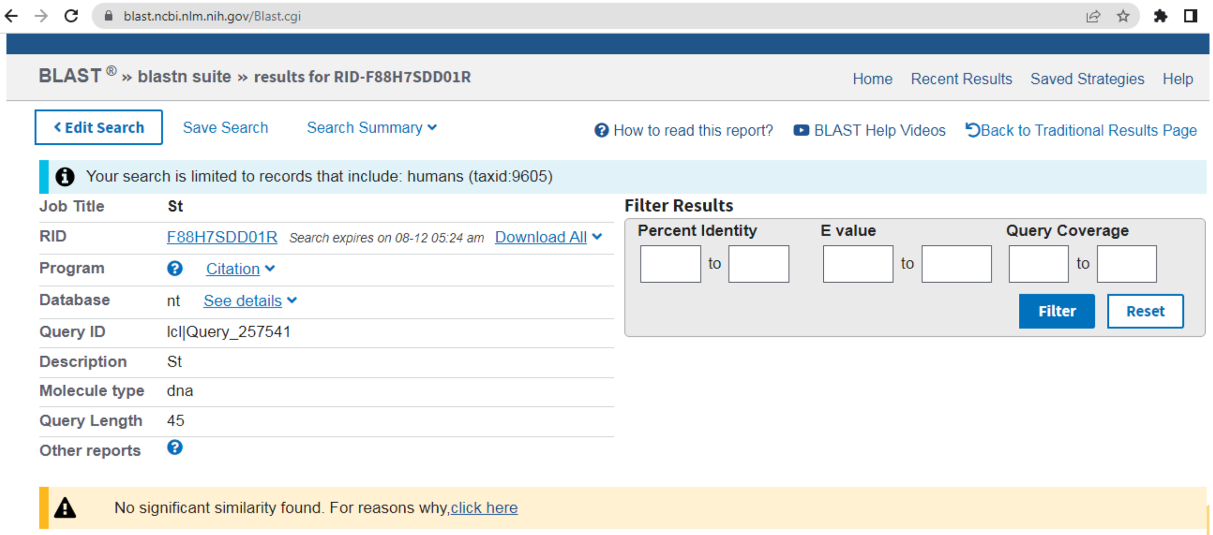

Analogous testing has been performed for all other human bacterial pathogens where the PreQ1 riboswitch is detected.

The last major BLASTn analysis concerned whether similar bacterial riboswitch sequences would be found in pathogenic and probiotic bacteria.

Figure S3. Blast analyses of PreQ 1 riboswitch and *Lactobacillus sp.*

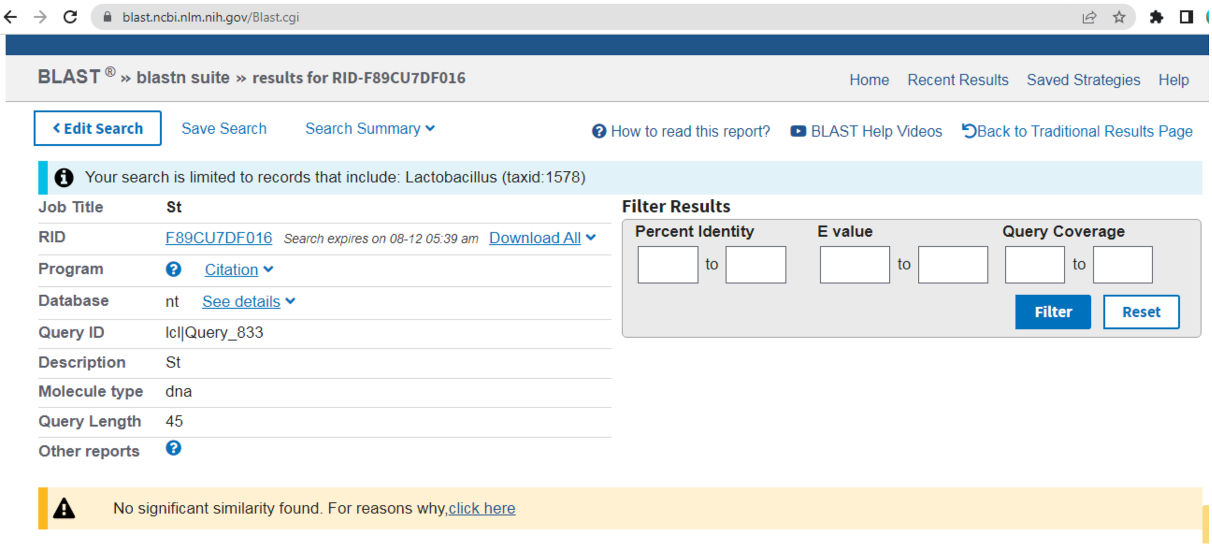

Following this protocol, all subsequent classes of riboswitches were analyzed. Some of the bioinformatics results have been added and are available to the RSwitch database, which is found on the official website of Prof. Penchovsky at the web address: https://penchovsky.atwebpages.com/applications.php?page=58.
